# Case Report: Expanding the Phenotypic Spectrum of Timothy Syndrome Type 1: A Sporadic Case With a *de novo* CACNA1C Pathogenic Variant and Segmental Ileal Dilatation

**DOI:** 10.3389/fped.2021.634655

**Published:** 2021-04-27

**Authors:** Ahmed A. Nugud, Nermeen Mahmoud ELkholy, Awad Alkarim Omar, Abid Qazi, Christos Tzivinikos, Nidheesh Chencheri, Sabina Khan, Muhammad Eyad Ba'Ath

**Affiliations:** ^1^Al Jalila Children's Speciality Hospital, Dubai, United Arab Emirates; ^2^Department of Clinical Sciences, Mohammed Bin Rashid University of Medicine and Health Sciences, Dubai, United Arab Emirates

**Keywords:** CACNA1C mutation, Timothy syndrome, Segmental Ileal Dilatation, syndactyly, long QT syndrome, seizure disorder

## Abstract

**Background:** Long QT syndactyly syndrome (long QT syndrome type 8), also known as Timothy Syndrome (TS) was first described in 1994 with still <50 case reported in the literature. The full spectrum of the syndrome is not yet known.

**Results:** Here we report a girl who presented with new onset refractory seizures and an undiagnosed cause of intermittent abdominal distention. She also had syndactyly of her fingers and toes and was found to have prolonged QT. Upon further investigations she was found to have a *de novo* pathogenic variant in *CACNA1C*, along with Segmental Ileal Dilatation (SID), and subsequently diagnosed with Timothy syndrome.

**Conclusion:** To our knowledge, the association of Timothy Syndrome with Segmental Ileal Dilatation, was not described before.

## Introduction

Timothy syndrome (TS), is an extremely rare genetic disorder, with an estimated incidence of one in a million, and <50 cases reported. TS was first described in the early 1990s, after a series of patients identified with arrhythmia disorder and syndactyly (1). The main mutation in TS affects the L-type calcium channel gene, and is usually inherited in an autosomal dominant fashion (2). The CACNA1C gene is expressed in many organs in the body, and its mutation leads to multiple systemic manifestations including; neurobehavioral issues, musculoskeletal deformities, and congenital heart defects (1). Other reported correlations with TS include gastroesophageal reflux, constipation, immune system impairment, and episodic hypoglycemia (1, 2). With the improvement in genetic testing modalities new mutations affecting CACNA1C gene have been described (3).

Here we report a *de novo* pathogenic variant in the *CACNA1C* gene in a patient with typical TS features and Segmental intestinal dilatation (SID), a previously undescribed feature of TS.

## Case Report

Female infant born on 34+1 week's gestation by emergency caesarean section for fetal distress. Birth weight 1,840 g and the parents were unrelated. The child was noted to have syndactyly of 3rd, 4th, and 5th fingers and 2nd, 3rd, and 4th toes bilaterally. Initially she required respiratory support which was weaned by 48 h of age. She was started on feeds on first day of life and by the 2nd day she was on full feeds. She developed severe abdominal distention on day 4 of life with feeding intolerance and vomiting, yet the child was still having bowel motions at the time. She did not respond to multiple colonic washouts and was taken to theatre on day 9 of life and had a reportedly negative exploration. Multiple biopsies were taken from colon to rule out Hirschsprung's Disease and later proved to be normal. The child initially did well after the surgery, as abdominal distention resolved and she was restarted on enteral feeds and they were well-tolerated, hence she was discharged home.

**Figure 1:**
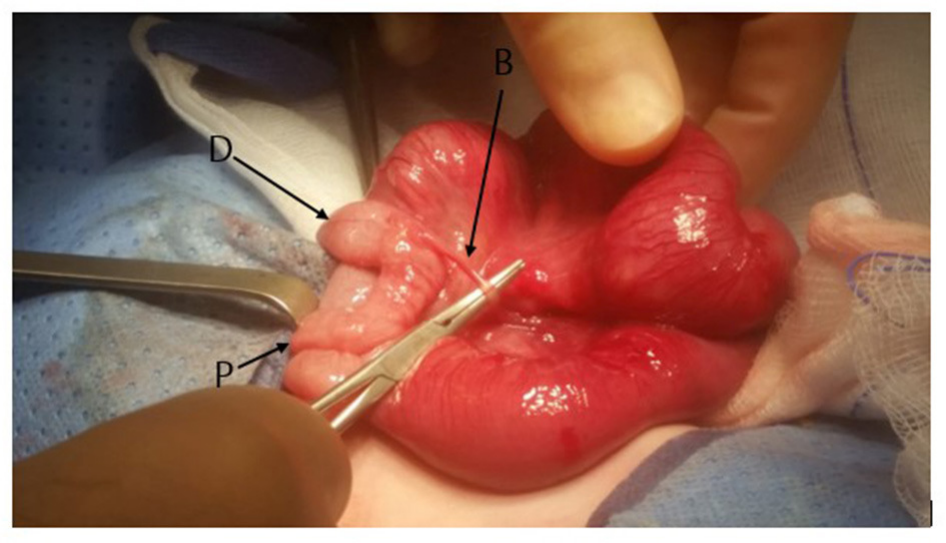
The appearance of the distal ileum at the time of original surgery. At the time SID was not identified. A band (B) attached to a Meckel Diverticulum can be seen. The proximal (P) and distal (D) caliber change is noted.

On day 27 of life the child presented to the emergency department with distended abdomen, and an emergency exploratory surgery to rule out adhesions was done, and a band was found across the ileum close to the ileocaecal valve, Figure 1. This band at the time was not thought to be obstructing. The child had creation of ileostomy at that level with a presumptive diagnosis of pediatric intestinal pseudo-obstruction.

**Figure 2:**
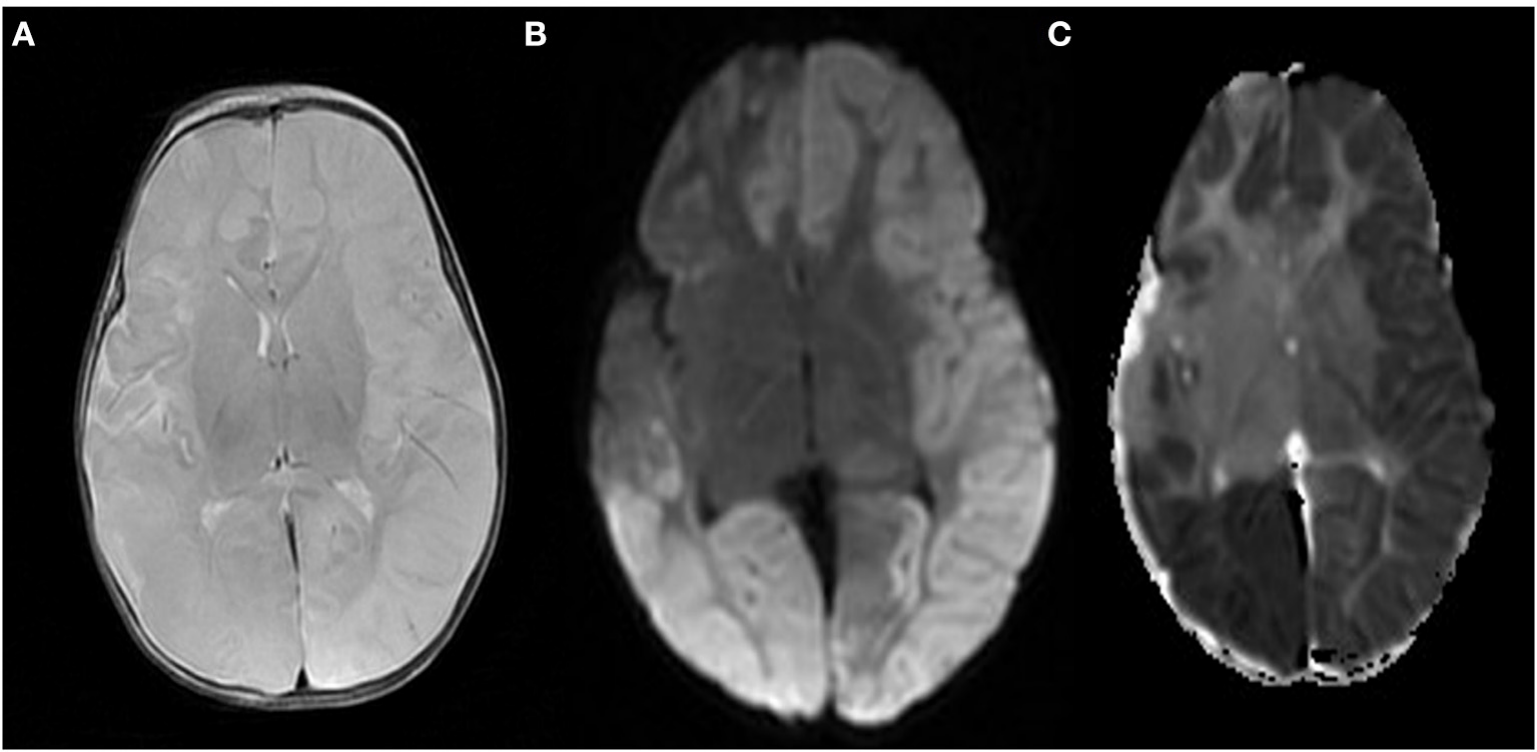
**(A)** MRI brain T2 weighted image that highlight cerebral oedema and cortical sulcal effacement. **(B,C)** Diffusion weighted image of the brain showing extensive areas of increased signal intensity with corresponding low ADC value, suggestive of hypoxic/ischemic brain injury.

Post-operative she exhibited excessive crying followed by abnormal movements in the form of clonic jerks of the upper limbs with twitching of eyes, this was associated with profound desaturation and tachycardia. Despite a loading dose of Levetiracetam and followed by a maintenance dose her seizures persisted. Thus she received a loading dose of midazolam followed by midazolam infusion. Following this the child was transferred to our facility at the age of 7 months with an ileostomy for further investigations and management of her seizures. Brain ultrasound was consistent with cerebral edema. With pediatric neurology input, the patient underwent an MRI brain which was reported to be consistent with diffuse cerebral edema, and extensive areas of restricted diffusion, Figure 2.

**Figure 3:**
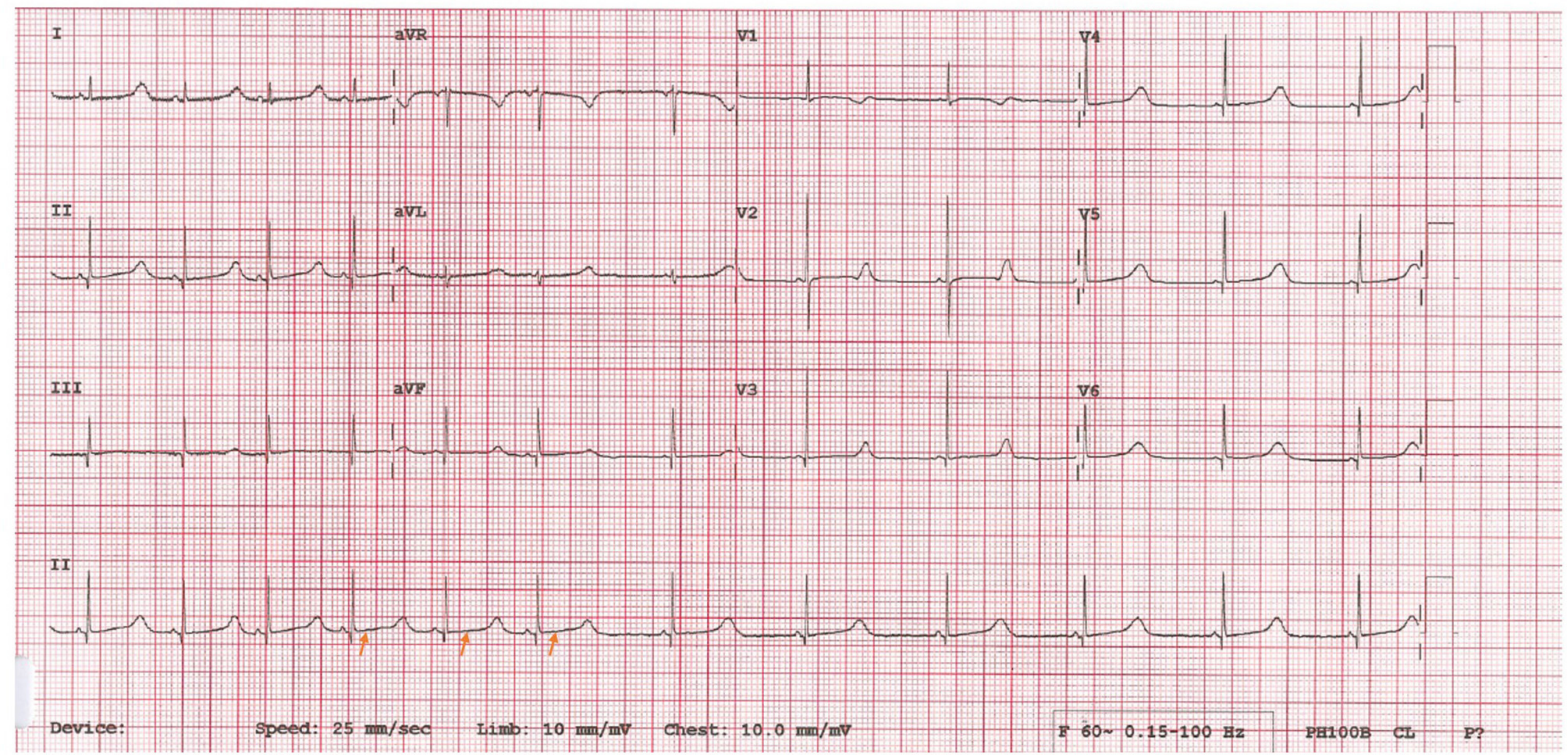
Twelve lead ECG showed sinus regular rhythm with Long QTC [QT = 0.52 s, QTC = 520. Prolonged] (arrows).

During her work up for seizures she was found to have prolonged QT on electrocardiogram which was then confirmed with 24 h Holter. She was started on propranolol 1 mg/kg/day (Figure 3). Given the association between syndactyly and prolonged QT interval; the possibility of Timothy Syndrome was considered. Trio (mother, father, and child) whole exome sequencing identified a heterozygous *de novo* missense variant in *CACNA1C* gene c.3061T>C; p.(Cys1021Arg), which was considered likely pathologic given the phenotype. Given the strong association with congenital heart anomalies, an echocardiogram was done which showed a small secundum atrial septal defect.

**Figure 4:**
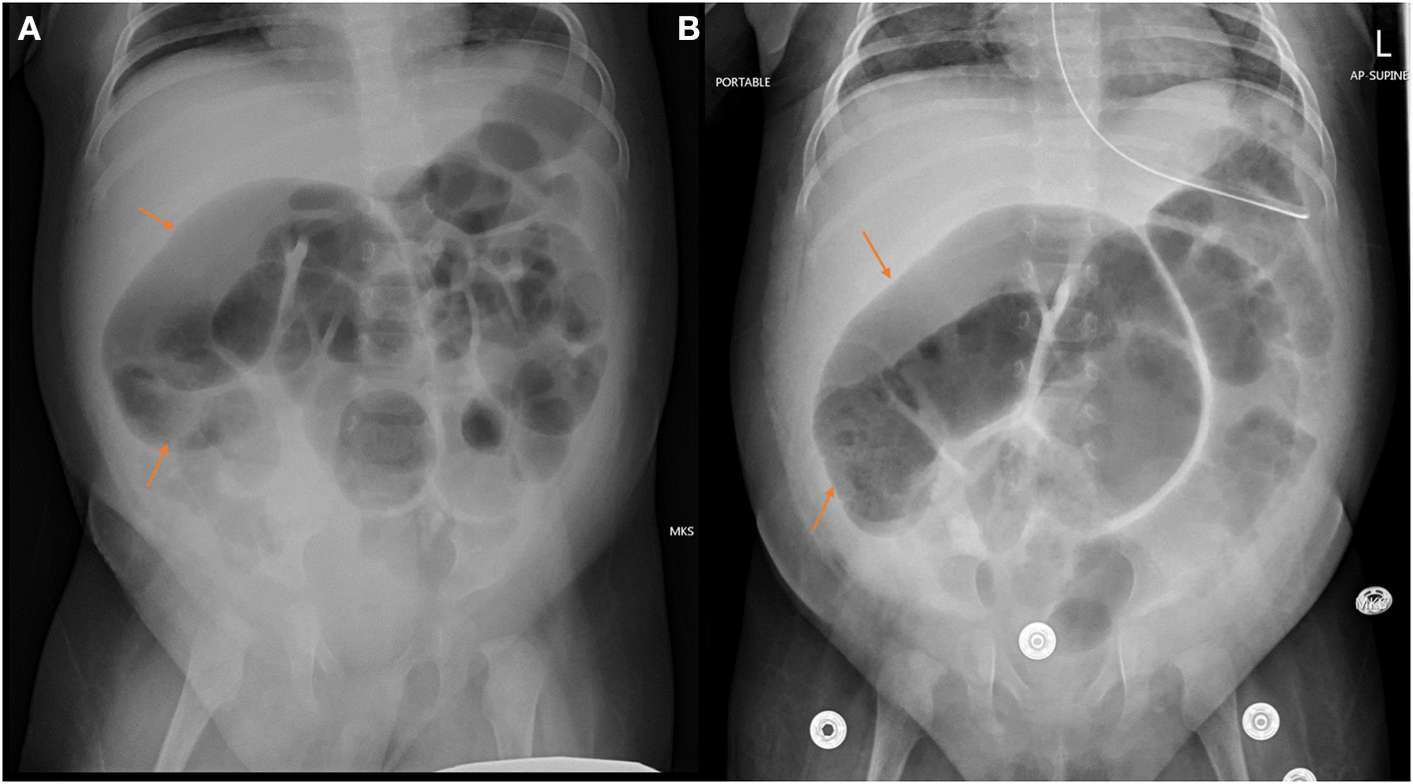
**(A)** Fixed loops of the dilated segments of ileum that became apparent within 24 h following ileostomy closure **(B)**.

During her hospital stay she was also troubled with high stoma output. She had a suction rectal biopsy and distal loopogram, both of which were normal. She then underwent ileostomy closure at age of 9 months. On the 3rd day post op she developed severe abdominal distension with markedly distended bowel loops that were fixed on serial x-rays (Figure 4). She was then re-explored and a long segment of distended and inflamed ileum proximal to the anastomosis was found and resected with redo primary anastomosis. The child did well following this surgery. Histopathology of the distended segment confirmed presence of ganglia. She was discharged home on full enteral feeds and continued to thrive well. She only had one episode of abdominal distension with PR bleeding and intestinal pneumatosis 1 month after the surgery that settled with conservative treatment. She is currently on laxatives for constipation as needed.

## Discussion

Splawski et al. (1, 2), described two distinctive groups of TS patients, and subsequently came to be known as TS type 1 and TS type 2, with main difference of presence or absence of syndactatly. Both groups had missense mutations affecting exon 8/8A, G406R in TS type 1 and G402S in type 2, of the gene encoding for CACNA1C, the L-type voltage-dependent Ca2þ channel (1, 2).

The main cause of death in TS is sudden cardiac death (SCD), likely due to fatal arrhythmias. Beta-blocker therapy has not been shown to protect against SCD (4, 5). Dufendach et al. (6), found that Nadolol conferred better protection against SCD compared to other beta blockers like propranolol. The authors also noted that most patient had better protection when medical therapy was combined with implantable defibrillator device (7).

Multiple reports highlight the high risk associated with anesthesia in patient with TS (6). Extreme peri-operative vigilance and avoidance of triggering factors can result in favorable outcome. Pre-operative management includes cardiac consultation and maintenance of adequate preoperative hydration. Cardiology involvement is of paramount importance for evaluation, optimization and pacemaker placement if the child is having recurrent bradycardic episodes. Beta-blocker therapy should be continued in the perioperative period although this may mask hypoglycemia and cause electrolyte imbalance (6, 7). A pre-operative basic metabolic panel or an arterial blood gas is of help to ensure normoglycemia and electrolytes especially potassium. Standard ASA monitors and two large bore peripheral intravenous catheters are adequate unless the surgery is extensive involving electrolyte and fluid shifts. In that case, arterial line and/or central line catheters are mandatory.

Sevoflurane; an inhalational agent is commonly used for induction and is reported to have a higher risk of SCD in TS patients compared to other anesthetic agents (6, 7). Alternatively, Propofol or thiopental can be used with extreme caution for induction. If a muscle relaxant is needed, cis-atracurium is ideal, however anticholinergic/anticholinesterase agents should be avoided for reversal (7). During critical anesthesia times such as induction, intubation, surgical stimulation, and extubation, measures should be taken to prevent sympathetic stimulation and autonomic dysfunction resulting in arrhythmias which could be fatal (8). Adequate analgesia can be obtained by utilization of lidocaine spray during intubation and pre-operative regional anesthesia (8). In case of arrhythmias, lidocaine, and magnesium bolus followed by infusion can be utilized.

Ventilatory settings should be such to maintain normocapnea and prevent increase in peak or end expiratory pressure (8). Valsalva maneuver should be avoided as it can prolong the QTc interval (8). Anti-PONV agents that can cause prolonged QTc interval such an anti-5HT3 antagonists (ondansetron) and anti-histamines should not be used, dexamethasone can be used instead (8). Our case underwent multiple operations under anesthesia, general and local, without significant complications with close cardiac physicians' involvement.

SID is a rare developmental anomaly that can affect any part of the gut, although, ileal involvement is the most common (8, 9). Around 150 odd cases of segmental intestinal dilatation are reported in literature but none of them provide any clues to the definite etiology of this disease (8). It is characterized by sharply demarcated dilatation of a gastrointestinal segment with clinical findings of intestinal obstruction or sometimes sub-acute obstruction, anemia, or malabsorption (9). The most often associated malformation is an omphalocele (8). Pathological examination may show the presence of heterotopic tissue like lung, pancreatic, esophageal, gastric, cartilage, and striated muscle in the dilated segment. Thinning or hypertrophy of the muscular layer has also been described (8, 9). Resection of the dilated segment and end-to-end anastomosis of the normal bowel is the definitive curative treatment.

In our case we believe SID was missed initially (appearance of the bowel at the time of initial surgery is shown in Figure 4) and then the formation of an ileostomy made the demarcation hard to recognize at the time of ileostomy closure. Only following ileostomy closure the full blown clinical and radiological presentation of SID became apparent and then subsequently appropriately treated. This delay in recognition of SID led to the child needing multiple surgeries and general anesthetics and could have been avoided.

To our knowledge the association between TS and SID has not been described before. It is possible that our case represents a random chance however the rarity of both conditions make this a remote possibility. More reported cases with ideally genetic work up are needed to clarify whether the association is true or not. Little is known about gastro-intestinal involvement in TS in general, however it is known that CACNA1C is expressed in the smooth muscles of the small intestine, yet it is not known if CACNA1C mutations can affect bowel motility in patients with TS (10). Dufendach et al. (6), reported a case with similar genetic mutation and clinical characteristics, but no SID was reported.

Our child still suffers with constipation despite the resection of significant length of bowel. It is not clear whether this represent an abnormality of bowel dysmotility. Our case suffered one episode of enterocolitis type symptoms with intestinal pneumatosis following her resection which again could be linked with abnormal bowel motility. In addition, she was also diagnosed few months after discharge with non –IgE mediated cow's milk protein allergy which responded to dairy free weaning and extensive hydrolysed formula.

## Conclusion

Segmental Ileal Dilatation and Timothy Syndrome are two rare conditions that do not seem to share a common etiology. We report the first association between them. More reported cases with genetic work up are needed to clarify whether this association is real or just a random chance.

## Data Availability Statement

The raw data supporting the conclusions of this article will be made available by the authors, without undue reservation.

## Ethics Statement

Written informed consent was obtained from the minor(s)' legal guardian/next of kin for the publication of any potentially identifiable images or data included in this article.

## Author Contributions

AN: literature search, literature review, wrote initial draft, and wrote subsequent drafts. NE and SK: literature search, literature review, wrote initial draft, and review of manuscript. AO: literature search, literature review, review of manuscript, and provided operation images and Loopogram. AQ: wrote subsequent drafts, review of manuscript, and provided operation images and Loopogram. CT: literature search, literature review, reviewed initial draft, wrote subsequent drafts, and review of manuscript. NC: literature search, literature review, provided brain radiological imaging, and review of manuscript. MB: wrote subsequent drafts, review of manuscript, provided operation images and Loopogram, and review of the final draft. All authors were actively involved in the patient care and have critically reviewed and approved the final draft and are responsible for the content and similarity index of the manuscript.

## Conflict of Interest

The authors declare that the research was conducted in the absence of any commercial or financial relationships that could be construed as a potential conflict of interest.
